# Protocol for a cluster randomised controlled trial comparing structured Follow-up And Monitoring Of new USers of NHS hearing aids to usual care: the FAMOUS trial

**DOI:** 10.1186/s13063-025-09188-9

**Published:** 2025-11-05

**Authors:** Kevin J. Munro, Christopher Armitage, Rachel Elliott, Gabrielle H. Saunders, Rebecca Haydock, Ted Leverton, Edmund Juszczak, Francesca Oliver, Christopher Partlett, Caroline Rick, Anne Schilder, Jane Wild, Paul Wilson, Magdalena Sereda, Michael Loughran, Grace Holt, Bethany Gill

**Affiliations:** 1 The University of Manchester, Manchester, UK; 2 Nottingham Clinical Trials Unit, The University of Nottingham, Nottingham, UK; 3 Royal National Institute for Deaf People, Peterborough, UK; 4 University College London, London, UK; 5 Betsi Cadwaladr University Health Board, Wales, UK; 6 NIHR Nottingham Biomedical Research Centre, Nottingham, UK

**Keywords:** Clinical trial, Cluster randomised controlled trial, Protocol, Hearing aid use, Follow-up, Behaviour change, Economic evaluation, Process evaluation

## Abstract

**Background:**

Hearing loss is a prevalent condition that impacts on social, mental and physical health, and has a significant economic burden. Hearing aids can improve the quality of life for those living with hearing loss; however, low and inconsistent use remains common. Within the National Health Service (NHS), follow-up care for new hearing aid users is highly variable and often lacks structure, which may contribute to low use. The FAMOUS trial investigates whether a structured care model for follow-up, combined with evidence-based behaviour change interventions, improves hearing aid use compared to usual care.

**Methods:**

FAMOUS is a multi-centre, two-arm parallel-group cluster randomised controlled trial (CRCT) with integral internal pilot, economic, and process evaluations. The trial involves 36 NHS audiology services and compares two types of follow-up for new adult hearing aid users: structured care, which includes personalised action plans, early monitoring, and routine follow-up at 6 weeks post-fitting, to usual care, which includes the offer of a follow-up 6–12 weeks after fitting. Recruitment is conducted through participating services over 3 months, with pseudo-anonymised routine data collected from electronic medical records of all patients who attend. Consent and outcomes are then collected from patients at 12 weeks post-fitting. For patients who provide consent to future contact, the primary outcome (self-reported daily hearing aid use) is collected at 12 months post-fitting. Secondary outcomes (quality-of-life (QoL), hearing-related disability, and economic measures) are collected at both timepoints. Qualitative interviews with a subset of patients and hearing professionals in the intervention arm will assess the acceptability and implementation of the intervention. Statistical analyses, including mixed-effects regression modelling, will be conducted under an intention-to-treat framework.

**Discussion:**

FAMOUS addresses a critical evidence gap regarding the potential benefits of follow-up care for new hearing aid users. If the intervention is successful, it can be rolled out nationally using existing facilities with limited impact on resources, identified in the economic analysis, and would improve hearing aid use and quality of life for those living with hearing loss.

**Trial registration:**

Prospectively registered with the International Standard Randomised Controlled Trial Number (ISRCTN) 10589817. Date of registration: 01/09/2022.

## Introduction

### Background and rationale {6a}

#### Background

Hearing loss is the leading cause of years lived with disability in the United Kingdom (UK) [1]. The prevalence of hearing loss is increasing: 12.5 million UK adults currently live with hearing loss, but it is estimated this will increase to 18 million by 2031 [2]. Hearing loss leads to communication difficulties between family, colleagues, and friends and affects well-being. It is associated with reduced QoL, depression and anxiety, poor social interactions and loneliness [3], with downstream effects arising from social isolation and possible cognitive decline [4] at a cost of £30 billion p.a. to the UK [5]. Partners (i.e. significant others) of people with hearing loss also experience frustration and anxiety resulting from communication problems [6]. Hearing aids are effective at improving hearing-related QoL for adults [7], as it is known that use correlates directly with benefit and satisfaction, yet research shows that, for those people fitted with a hearing aid(s), about 30% use them only some of the time, and a further 20% do not use them at all [4].

The direct cost to the NHS of managing hearing loss is £450 million per annum (p.a.) [8]. The NHS is the largest purchaser of hearing aids in the world, procuring around 1.2 million p.a. with around 355,000 new adult patients p.a. However, low and non-use can have consequences for:

i. Hearing aid wearers and their families: non-users receive no benefit [4],
ii. Potential hearing aid wearers: negative perceptions may discourage or delay help-seeking [4],
iii. Society: economic consequences of untreated hearing loss are vast, yet non-use of hearing aids may lower their perceived effectiveness and weaken the case for securing scarce resources [4].

Addressing low hearing aid use was identified as one of the top five research priorities by the James Lind Alliance priority setting partnership [9].

#### Rationale

It is well-established that monitoring and follow-up have consistent positive effects for long-term adherence to health interventions [10]. Within the NHS, monitoring and follow-up for adults prescribed a hearing aid(s) for the first time are variable and ill-defined [11] and patients generally do not initiate follow-up consultations to resolve problems [12]. A survey completed by NHS audiologists from across the UK (when preparing for this trial) showed that follow-up appointments are not always offered, and when they are offered, content is highly variable, and the timing ranges from 6–12 weeks after the hearing aid(s) are fitted [10].

The National Institute for Health and Care Excellence (NICE) adult hearing loss guideline committee carried out a systematic review on the benefits of monitoring and follow-up in new adult hearing aid users [10]. No relevant studies were identified, which likely contribute to the wide variation in practice across the UK. Studies investigating reasons why people cease using their hearing aids have identified easily resolvable issues [13, 14], including discomfort in the ear, own-voice sound quality, and uncomfortable loudness. Recognising that adults with hearing aid issues will likely use them less [15, 16] and do not initiate follow-up [12] and that evidence regarding the effects of follow-up is lacking, the NICE guideline committee recommended a randomised controlled trial (RCT) of structured follow-up and monitoring as a high priority.

FAMOUS (*F*ollow-up *a*nd *m*onitoring *o*f new *us*ers of NHS hearing aids) has been designed to assess whether a 4-step follow-up and monitoring intervention will increase self-reported daily hours of hearing aid use in first-time adult hearing aid(s) users, 12 months after initial fitting. By addressing this urgent unmet need, FAMOUS will evaluate QoL and health outcomes for adults with hearing loss and their families, potentially providing cost savings to the NHS and society. The intervention is based on current evidence and has been refined by and co-developed with input from NHS audiologists and our patient public involvement (PPI) groups to ensure it is fit for purpose in a UK-wide setting. If the intervention is successful, it can be rolled out nationally using existing facilities with a presumed limited impact on resources, that will be confirmed in the economic analysis.

### Objectives {7}

The primary objective is to compare self-reported daily hours of hearing aid use 12 months after initial hearing aid(s) fitting, in adults offered hearing aids for the first time who receive the FAMOUS structured care intervention, to that reported by adults who receive usual care.

### Trial design {8}

**Fig. 1:**
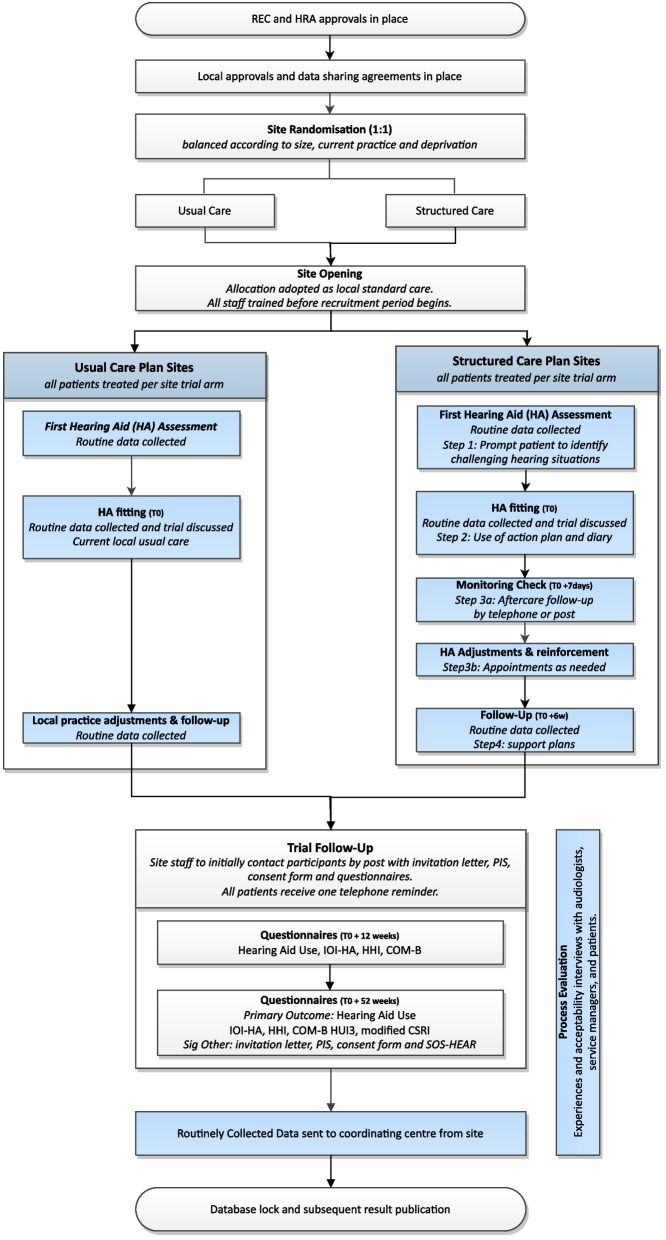
Patient care pathway

FAMOUS is a multi-centre, two-arm parallel-group CRCT with integral internal pilot, economic and process evaluations. During a 3-month recruitment phase at each participating NHS adult hearing service, the service’s trial allocation (structured care or usual care) will be adopted as their sole clinical follow-up plan. All patients attending the participating services for a hearing assessment will be treated in line with the service’s trial allocation and will subsequently be invited to complete patient-reported outcome measures (PROMs) 12 weeks after their hearing aids have been individually prescribed, fitted and verified. Those who consent and provide outcomes at 12 weeks after fitting will be re-contacted at 12 months for collection of the primary outcome. The trial design is detailed in Fig. 1.

The target population is adults (≥ 18 years) who present with hearing difficulties and are offered conventional, acoustic NHS hearing aids for the first time. All eligible patients who attend for a new assessment appointment at a participating NHS hearing service during the recruitment phase will be informed about the trial by their hearing professional (including but not limited to, audiologists, audiology assistants, or audiology associates). Trial posters with a link to the trial website will be displayed in clinic and waiting room areas to provide further information about the trial. Routine pseudo-anonymised medical data will be collected from all eligible patients, including those who decline a hearing aid(s) fitting.

Patients in both arms of the trial will receive usual NHS care for the prescription and fitting of a new hearing aid [10]. This comprises:

1. An initial hearing assessment appointment during which there is:
  i. A case history and examination,
  ii. Assessment of hearing and communication needs, and,
  iii. Assessment of hearing loss.
2. A fitting appointment during which:
  i. The output of the hearing aid(s) is measured in the patient’s ear to ensure it is delivering the prescribed amplification,
  ii. The patient is provided with information about the benefits and challenges of using hearing aids, technical information about how the hearing aid(s) works, e.g. location of the microphone, function switches and guidance on when to use the hearing aid(s) which, for the majority of people, will be for most of the day,
  iii. The healthcare professional gives a practical demonstration as to how to use the hearing aids, e.g. change batteries, fit into ear, and,
3. A follow-up appointment to:
  i. Evaluate the benefit(s) the patient is receiving from the hearing aid(s),
  ii. Address ongoing concerns, difficulties, and identify any unmet additional needs requiring further intervention or onward referral,
  iii. Reinforce information provided at the time of fitting,
  iv. Provide additional information, e.g. about support services and hearing aid features, and,
  v. Make necessary adjustments to the hearing aid(s).

Structured care: Patients attending clinics randomised to the intervention arm will, in addition to receiving usual care described above, receive a follow-up and monitoring behaviour change intervention delivered by their hearing professional, which comprises four steps considered to be ‘best practice’ for a new adult hearing aid user, as described in 11a. The steps in this pathway are personalised to the patient, aimed at addressing their individual needs and creating individualised plans to overcome problems with their hearing aid(s) difficulties. Personalised health care helps people make decisions about managing their health so they can live the life they want to live based on what matters to them, working alongside clinical information from the professionals who support them. Evidence shows that people will have better experiences and improved health and wellbeing if they can actively shape their care [17, 18].

Usual care: The control group will receive usual NHS care as outlined above. The follow-up according to NICE guidelines should be the offer of an appointment 6–12 weeks after the initial hearing aid(s) fitting and should be face-to-face unless the patient prefers an alternative approach (i.e. telephone call). Since the COVID-19 pandemic, many services have moved to remote contact (i.e. telephone follow-up), which is acceptable for the FAMOUS trial. Services that are not routinely offering a follow-up for new adult hearing aid users will not be able to participate in FAMOUS.

The trial contains an internal pilot 6 months after the first site opening, with stop/go criteria set at levels, which if maintained and the progress approved by the Funder, will enable completion of the remaining trial activities. Details of the internal pilot are described in 21b. The trial will contain a Study Within A Trial (SWAT) to investigate whether the timing of a reminder telephone call impacts the return rate of outcomes at 12 weeks post-fitting. Further details on the SWAT can be found in the online SWAT Store (SWAT191) [19].

## Methods: participants, interventions and outcomes

### Study setting {9}

The trial is conducted across 36 NHS-funded adult hearing aid services across the UK. A list of the participating services can be found on the trial website (www.famousstudy.ac.uk).

### Eligibility criteria {10}

#### Inclusion criteria

Adults (≥ 18 years); being offered hearing aids for the first time.

#### Exclusion criteria

Adults offered an auditory implant of any kind; offered non-conventional hearing aids, e.g. that reroute sound between ears.

### Who will take informed consent? {26a}

Individual written consent is sought from patients for completion of the follow-up research questionnaires at 12 weeks and 12 months after hearing aid(s) prescription and fitting. This approach has been approved by ethics and was recommended by the Confidentiality Advisory Group (CAG), who advised explicit consent was required to confirm an individual’s participation in the trial. Consent will also be sought from the participant’s Significant Other if they identify one, as part of the collection of the secondary outcome including third-party disability experienced by close relations of those with a hearing loss. Participants can opt to take part in a semi-structured interview when completing their initial consent to join the trial; for those who do take part in the semi-structured interview, verbal consent to participate will be obtained by the Research Associate conducting the interview.

Given the trial is cluster rather than individually randomised, there is no requirement to obtain consent at baseline when patients attend their hearing assessment appointment; all eligible patients are automatically enrolled into the follow-up pathway to which the audiology service has been randomised. This is because services in both arms of the trial are delivering standard NHS care, with the intervention comprising elements considered to be ‘best practice’. Patients will not be given a choice of the follow-up they receive after they have their hearing aid(s) fitted, and so they will not opt in or out of the follow-up pathway to which the site has been randomised. Obtaining informed consent in clinic could also mean patients may decline the prescription of the hearing aid(s), believing that the hearing aid prescription and fitting is primarily for research purposes and not for their clinical need. By not taking up clinic time to obtain informed consent, the burden on healthcare professionals during clinic appointments will be reduced and also reduces biased selection of participants by hearing professionals (overtly or unintentionally due to time pressures).

### Additional consent provisions for collection and use of participant data and biological specimens {26b}

The participant consent form will include optional consent for the sharing of patient details with a third party for coordination of reminders for questionnaire completion (by text message).

## Interventions

### Explanation for the choice of comparators {6b}

The control group will receive usual care at the hearing assessment, and prescription and fitting appointments, as described in Section Trial design {8}.

### Intervention description {11a}

The FAMOUS intervention is based on best practice across the NHS and is designed to increase uptake and use of hearing aids. The intervention will deliver personalised care using behaviour change approaches and will promptly address potential problems that the hearing aid user may be experiencing. These are all examples of best practice that are not limited to adult hearing aid services. The intervention comprises four steps:

i. At the initial hearing assessment appointment: Patients, in consultation with their hearing professional, will identify situations in which they would like to hear better so that their management can be personalised. This activity is consistent with the Client Oriented Scale of Improvement (COSI) outcomes measurement [20].
ii. At the hearing aid prescription and fitting appointment: A ‘Hearing Aid User Checklist and Diary’ booklet will be provided to all patients to assist them with self-monitoring their hearing aid use and experiences of common problems over the first 12 weeks, based on the situations identified during the assessment appointment. A personalised action plan will also be developed by the patient in collaboration with their hearing professional to help reinforce where and when their hearing aid(s) should be used, and to help integrate them into daily life. The action plans created by the patient will be ‘when-then’ plans, also known as implementation intentions [21]. Patients will create these plans using scenarios that form a part of their everyday life (e.g. when I go into the kitchen to make my morning cup of tea, then I will put in my hearing aid(s)’.
iii. Seven-day post-fitting contact by telephone: Monitoring of progress at 7-days after prescription and fitting in all patients to assess hearing aid use and any problems encountered with the aim of reducing low hearing aid use. Patients with low hearing aid use (i.e. used for less than 1 hour each day) and/or experiencing significant problems that are preventing them from wearing their hearing aid(s) as planned (i.e. discomfort) will be invited back to the clinic. Once they return to clinic, the diary and action plan will be reviewed by the hearing professional who will offer solutions to their problems.
iv. Six-week follow-up after the hearing aid prescription and fitting appointment: This will be offered as a face-to-face appointment where possible, or can be remote (telephone, video consultation) if the patient prefers an alternative approach. In addition to the usual care provided, as detailed in Section Trial design {8}, hearing professionals and patients will review the action plans that were made at the fitting appointment and will reiterate and reinforce the plans if the patient is managing well with their hearing aid(s). New action plans can be created if the previous ones are no longer relevant (e.g. the patient’s daily routine has changed). If needed, ‘coping’ and/or ‘support’ plans can be created to help the patient overcome any problems they might encounter with their hearing aid(s).

### Criteria for discontinuing or modifying allocated interventions {11b}

Patients are encouraged to follow all four steps of the structured care pathway listed in 11a and complete weekly tasks within their Hearing Aid User Checklist and Diary at home, in between their hearing aid prescription and follow-up appointments, and alongside any additional investigations and treatment regarding their hearing aid care. Modification or discontinuation of the structured care pathway is at the discretion of the hearing professional.

### Strategies to improve adherence to interventions {11c}

To improve hearing professionals’ adherence to the additional steps in the trial intervention, supplementary materials are provided to intervention sites to remind staff of the steps included in the pathway and tasks to be completed at each clinic appointment. This includes a table of key tasks, a traffic light grading system to support the 1-week telephone follow-up, and posters to prompt hearing professionals to faithfully follow the four key steps of the intervention. Adherence to the intervention is monitored monthly by the Trial Management Group (TMG). Protocol adherence is reported to the TMG at site level and patient level, using data provided in the routine uploads from sites. Sites will be considered as having adhered to the protocol if 80% of patients have received the key steps of the intervention, as defined in point 2 of our pilot criteria (see Section Interim analyses {21b}). Additional support will be offered to services if there are concerns about adherence to the intervention.

### Relevant concomitant care permitted or prohibited during the trial {11d}

Patients may be offered concomitant care in relation to their hearing loss if required, alongside the FAMOUS follow-up pathway their NHS audiology service has been randomised to.

### Provisions for post-trial care {30}

Patients will remain under the care of their audiology clinic for hearing aid follow-up and ongoing maintenance and support.

### Outcomes {12}

The primary outcome is self-reported daily hours of hearing aid use 12 months after the hearing aid(s) fitting. These data will be obtained by having the patient self-report ‘On a typical day over the last week, how many hours did you use your hearing aid(s)?’ The data will be collected via participants’ preferred contact method (post, telephone, or text); this approach avoids the requirement for hearing aid users to return to the clinic and should result in less attrition.

Secondary outcomes collected at 12 weeks and 12 months are:

i) Self-reported daily hours of hearing aid(s) use.
ii) International Outcome Inventory for Hearing Aids (IOI-HA), which is a seven-item questionnaire designed to be generally applicable in evaluating the effectiveness of hearing aids. There are questions on usage, benefit, residual difficulties, satisfaction, QoL, impact on others and worth the effort. Participants can select from five pre-defined answers which are assigned a value of 1 to 5, with higher scores indicating a more favourable outcome.
iii) Hearing Handicap Inventory (HHI), which is a 22-item survey with questions on hearing-related QoL. Participants can select from three categories (Yes/Sometimes/No) for each question, which assesses how an individual perceives the social and emotional effects of their hearing loss. Higher scores indicate a worse outcome.
iv) Capabilities, Opportunities, Motivations and Behaviour (COM-B) is a six-item questionnaire that measures capabilities, opportunities, and motivations to engage in a behaviour change, i.e. wearing a new hearing aid(s). Participants can score how much they agree with a statement from 1 to 10, with a higher score indicating a better outcome.
v) The Significant Other Scale for Hearing Disability (SOS-HEAR) is a 27-item questionnaire that will be completed at 12 months by the participant’s partner, if they can identify one. This will measure the third-party disability, with partners scoring the severity of a problem from 0 to 4. A higher score indicates greater difficulties for the significant other.
vi) Effects are captured at the individual patient level through calculating quality-adjusted life years (QALYs) using Health Utilities Index 3 (HUI-3) at 12 months post-hearing aid(s) fitting. HUI-3 is a 17-item questionnaire that participants can rank their ability to do something with between four and six severity options. A higher score indicates the worst possible health state.
vii) A broader understanding of the effect of hearing loss on wider costs will be measured using the modified Client Service Resource Inventory (CSRI). This tool is used to collect information on the range of NHS services the new hearing aid user has utilised in their first year of hearing aid use, to estimate the cost per patient to the NHS.

Qualitative assessment of the trial:

Semi-structured interviews will be conducted to explore the experience and acceptability of the intervention and usual care. After 12 weeks of hearing aid(s) use, patients (*n* = 60) who have consented to take part in the trial and be contacted regarding an interview will be invited to be interviewed by the Research Associate to explore experiences and acceptability of the trial and trial intervention. Participants will be selected by the Research Associate from an equal mix of control and intervention sites, based on participant hearing aid use. Participants will be categorised according to their self-reported daily hours of use/non-use based on their answer to the ‘Hours of Daily Use’ question at 12 weeks post fitting. The categories are those who use their hearing aid/s for the following: (i) most of the day (8 h or more); (ii) part of the day (more than 4 h but less than 8 h); (iii) a small part of the day (more than 1 h but less than 4 h); or are not using their hearing aid/s at all on most days. Interviews will also take place with hearing professionals in the Structured Care arm (i.e. service managers and clinicians within the audiology department) and will be conducted at two time points: (i) while the intervention is taking place, to focus on perceptions and attitudes, training, and reflections on initial implementation experiences, and (ii) when the intervention is complete, to focus on the barriers and enablers to integrating the FAMOUS intervention within existing management care pathways.

### Participant timeline {13}

**Fig. 2:**
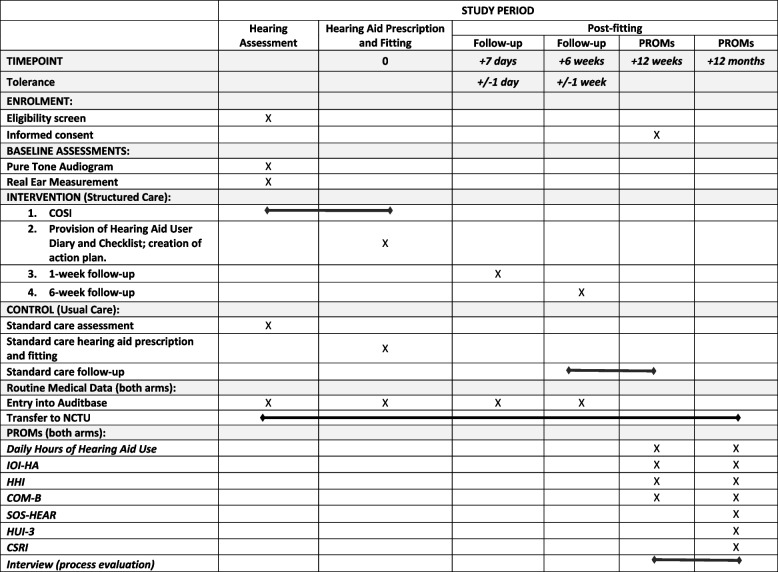
FAMOUS SPIRIT schedule of enrolment, interventions and assessments

The participant timeline is detailed in Fig. 1. The participant schedule of enrolment, interventions and assessments is detailed in Fig. 2 SPIRIT figure. Any new adult who attends a hearing assessment appointment at a participating adult hearing aid service during the 3-month recruitment period will be included in the trial. All patients who receive a hearing aid(s) will then follow the clinical pathway to which their audiology service has been randomised, as described in Section Trial design {8}. All patients who receive a hearing aid(s) will be invited to complete a research questionnaire 12 weeks after their hearing aid(s) fitting. If a patient completes their consent form and 12-week questionnaire, they will be sent another questionnaire at 12 months post-fitting for the collection of the primary outcome data. A subgroup of participants who provide consent to take part in a semi-structured interview will be contacted after 12 weeks of hearing aid(s) use.

### Sample size {14}

There are approximately 140 traditional NHS services (supplemented by some High Street providers funded to support the NHS) fitting hearing aids to 355,000 new adult users each year, so approximately 211 per service each month. We assume that 25% of participants will provide individual follow-up research data at 12 weeks post-fitting, with 80% of these providing primary outcome data (i.e. 20% of all patients enrolled in clinic). Since services will each recruit for 3 months, this will give an average cluster size for analysis of approximately 130 participants. The intra-class correlation coefficient (ICC) for the primary outcome is unknown, but based on published ICC data for a broad range of outcomes and settings, we assume it to be between 0.02 and 0.05 [22].

Our target treatment effect is a difference in mean hours of use per day of 1–1.5 h. With 90% statistical power, a 5% two-sided significance level, ICC = 0.02, a standard deviation = 5.5 [23] hours, and a target mean difference of 1 h, a total of 36 sites and 4680 participants are required for the analysis. Based on a 25% consent rate (at 12 weeks), a total of 23,400 patients need to be recruited, with 5850 participants consenting to follow-up data collection at 12 weeks post-fitting and 4680 providing primary outcome data at 12 months.

If the ICC is 0.05, the trial will have 90% power to detect a difference of 1.4 h (84 min), and 80% power for a difference of 1.2 h (72 min). There is an association between daily duration of hearing aid use and reported benefit, such that even a small effect of the intervention in increasing mean hours of hearing aid use could be clinically important. Our PPI contributors indicated that 60–90 min is a meaningful increase in usage.

Table 1 below shows the detectable differences in mean minutes of hearing aid usage at 80% and 90% power for ICCs ranging from 0.02 to 0.05 and coefficient of variation from 0.4 to 0.9.

**Table 1:** Detectable differences in mean for minutes of hearing aid usage with varying power, intra-class correlation and coefficient of variation

		**Intra-class correlation coefficient (ICC)**			
		0.02	0.03	0.04	0.05
**Coefficient of variation (CoV)**		90% power			
	0.4	60	70	79	86
	0.5	61	70	79	86
	0.6	61	71	80	87
	0.7	62	72	80	88
	0.8	64	73	81	89
	0.9	65	74	82	89
		80% power			
	0.4	52	61	68	74
	0.5	52	61	68	75
	0.6	53	61	69	75
	0.7	54	62	70	76
	0.8	55	63	70	77
	0.9	56	64	71	77

### Recruitment {15}

Patients will be informed and reminded about FAMOUS during their clinic visits and told to expect the 12-week questionnaire pack in the post. To supplement this information, and to ensure the trial remains as inclusive as possible, an animated video about the trial and what patients should expect will be available in four key languages spoken in the UK (English, Polish, Punjabi and Welsh). Links to the videos are provided (on trial posters and information cards) and on the trial website. Patients will receive a single telephone call to remind them to complete their 12-week questionnaire. As a ‘thank you’ gesture, all participants will be given a £10 shopping voucher for completing research outcomes at the 12-week and 12-month time points.

## Assignment of interventions: allocation

### Sequence generation {16a}

Randomisation will take place at a cluster level (NHS audiology service) with a 1:1 allocation ratio to structured care or usual care, using stratified block randomisation (stratifying by tertile of site size determined by obtaining up-to-date figures on the number of new hearing-aid fittings per month directly from participating services before randomisation). The trial statistician will prepare a dummy randomisation list using Stata version 17 [24]. The trial database systems developer will modify the random seed to generate the real randomisation schedule. There is a second-level randomisation for the SWAT.

### Concealment mechanism {16b}

NHS audiology services will be randomised after confirmation of their participation via a centrally held concealed allocation list hosted by the Nottingham Clinical Trials Unit (NCTU). The Trial Manager (or authorised designee) will access the NCTU web-based randomisation system to obtain the allocation for the site.

### Implementation {16c}

Site teams will be notified of their randomisation allocation via email prior to Site Initiation Visit (SIV), and subsequently trained according to their randomisation allocation. Site teams include clinical hearing professionals within the audiology department and research and development staff who will be responsible for delivery of the trial protocol.

## Assignment of interventions: blinding

### Who will be blinded {17a}

It is not possible to blind the intervention.

### Procedure for unblinding if needed {17b}

N/A.

## Data collection and management

### Plans for assessment and collection of outcomes {18a}

#### Routine medical data

Baseline clinical data will be extracted from Auditbase, the audiology electronic health record and patient management system used by NHS audiology services across the UK. Recruiting sites will install a custom script that will produce a report containing the baseline pseudonymised clinical data required for the trial. This includes demographics (age, sex and ethnicity), hearing assessment (audiogram information, real-ear verification of the prescription), hearing aid prescription (model and manufacturer, dates of issue) and the frequency and format of clinic appointments. All data is collected at baseline, during the assessment and fitting appointments, except for follow-up clinic appointments over the first year of hearing aid use; this data is collected each time a new report is run and provided to the NCTU.

#### PROMs

After 12 weeks of hearing aid use, eligible patients will be sent a paper questionnaire pack by their local audiology team. This will include a consent form and a questionnaire booklet for participants to complete themselves, either on paper or electronically, which can be accessed via a quick-response (QR) code. Paper questionnaires will be returned by post to the trial team at the NCTU, using pre-paid stamped addressed envelopes supplied within the questionnaire pack. Participants who consent to be contacted again at 12 months post-fitting will be sent a second questionnaire pack via their preferred method (email or post) as specified on their consent form. Data collection forms can be found in the electronic Trial Master File (TMF).

#### Qualitative trial data

Participants, hearing professionals and service managers will be contacted directly by telephone to arrange interviews. Interviews will be conducted via telephone or video call, and audio recorded, anonymised, and transcribed for analysis.

### Plans to promote participant retention and complete follow-up {18b}

During clinic appointments hearing professionals will encourage patients to complete and return their 12-week questionnaire. They will be informed that they will receive a £10 shopping voucher if they complete and return their questionnaires. When completing their consent form at 12 weeks post-fitting, participants can opt in to receive up to three text reminders to complete their 12-month questionnaire. Participants who do not return their 12-month questionnaire after three reminders have been sent will receive a telephone call from the trial team, and they will be asked for the primary outcome measure.

In order to maintain participant interest, updates about trial activity and relevant information about hearing will be posted on a trial website and shared via social media.

### Data management {19}

All trial baseline data are part of core clinical data held within Auditbase. The Principal Investigator (PI) or delegated hearing professional will be responsible for running the FAMOUS custom report within Auditbase to extract the routine data required for the trial. A member of the site study team will then upload the baseline clinical data reports into the FAMOUS Research Electronic Data Capture (REDCap) database monthly.

Routine clinical data from Auditbase is required from first patient first visit (hearing assessment) up to 12 months post-fitting of the last patient enrolled (last patient first visit + 12 months). Patient data collected from Auditbase will be pseudo-anonymised using Auditbase identifier (ID) and Site ID so that the patient cannot be identified by anyone outside of their direct care team. At 12 weeks post-hearing aid fitting, the questionnaire pack sent to patients will be labelled with the same Auditbase ID and Site ID number. Upon receipt of the returned consent form and questionnaire pack, the two datasets will be linked using the Auditbase ID and Site ID. Data linkage is verified by ensuring the date of birth provided by the participant matches the month and year of birth provided in the Auditbase reports. Any discrepancies are queried with the site staff. Patient identifiable contact information will be collected in the 12-week questionnaire pack with consent, and this will then be used by the FAMOUS trial team to re-contact the participant at 12 months post hearing aid fitting and for the qualitative interviews, where applicable.

Paper questionnaires returned to the FAMOUS trial team at NCTU will be entered into the FAMOUS REDCap database by a member of the NCTU data management team, and quality reviewed by a separate member of the team. Decisions on how to treat anomalous data will be made by members of the TMG blinded to allocations and documented in the Data Management Plan (DMP) and/or Statistical Analysis Plan (SAP) (where required).

### Confidentiality {27}

The FAMOUS REDCap database will be held in a secure server hosted by the University of Nottingham.

Pseudo-anonymised routine clinical data will be accessed only by the FAMOUS trial team for the purposes of monitoring and data linkage. The FAMOUS data and programming teams are responsible for linking routine clinical data with questionnaire data. The anonymised dataset will be saved on password-protected machines only accessible to members of the trial team at NCTU. Identifiable information about participants (such as address data for questionnaires) will be held in a separate area to the pseudo-anonymised linked dataset with access restricted to those involved in follow-up only, as authorised by the Chief Investigator (CI). Personal data recorded on all documents will be regarded as strictly confidential and is handled and stored in accordance with the Data Protection Act 2018. Identifiable patient contact details will be deleted prior to database lock and will not be included in the study analysis.

### Plans for collection, laboratory evaluation and storage of biological specimens for genetic or molecular analysis in this trial/future use {33}

Not applicable.

## Statistical methods

### Statistical methods for primary and secondary outcomes {20a}

The analysis and reporting of the trial will be in accordance with Consolidated Standards of Reporting Trials (CONSORT) guidelines for cluster trials [25]. A full SAP has been developed and will be finalised prior to database lock and agreed with the Trial Steering Committee (TSC).

The primary approach to between-group comparative analyses will be by intention-to-treat (i.e. including all patients according to randomised allocation regardless of site adherence to trial allocation). The main analysis for the primary outcome will include all randomised participants who consent to provide follow-up data at 12 weeks or 12 months. Where appropriate, imputation will be used so that all randomised participants may be included in a sensitivity analysis.

Descriptive statistics will be used to describe balance between the groups at baseline at site- and patient-level [26], including the characteristics of those that do and do not provide consent to follow-up. The primary comparative analysis will employ a mixed effects linear regression model to compare the hours of use at 12 months in each group, adjusting for factors balanced at randomisation and participant-level characteristics (e.g. age, sex, socio-economic status), where technically possible. The model will include a random effect to adjust for clustering within sites. The comparison will be presented as a difference in means, along with a corresponding 95% confidence interval.

Secondary outcomes will be analysed using appropriate multilevel regression models dependent on data type (e.g. binary, continuous, time-to-event), adjusting for factors balanced at randomisation and participant-level characteristics (e.g. age, sex, socio-economic status), where technically possible. The model will include a random effect to adjust for clustering within sites.

Appropriate interaction terms will be included in the primary regression analyses to conduct subgroup analyses according to the severity of hearing loss at baseline, sex, index of multiple deprivation, and unilateral/bilateral hearing aid fitting, but this analysis will be regarded as exploratory as the study is not powered to detect interactions. Interpretation of any subgroup effects will be based on the treatment-subgroup interaction and the corresponding 95% confidence interval.

### Interim analyses {21b}

No formal interim analyses are planned. The trial includes an internal pilot with stop/go criteria set at levels, which, if maintained, will enable trial completion as per the monitoring plan. This will be reviewed by the trial Data Monitoring Committee (DMC) 6 months after the first site green light;

i. Feasibility of recruiting sites: 24 of the 36 sites should have opened.
ii. Sites’ adherence with delivering intervention: 9 sites should have delivered at least 1 month of the intervention to all eligible patients. Adherence to the structured care intervention will be measured using the routine data collected from sites. Sites will be considered as adhering to the intervention if 80% of new hearing aid patients receive the below steps in the intervention:
  Step 1: COSI Part 1 completed at hearing assessment
  Step 2: FAMOUS Hearing Aid User Checklist and Diary provided to patient, action plan completed, and patient shown how to utilise the checklist and diary
  Step 3: Contact from their hearing service 7-days post-fitting by telephone
iii. Recruitment into the trial: 300 participants should have completed the 12-week patient-reported outcomes.

### Methods for additional analyses (e.g. subgroup analyses) {20b}

#### Health economic analysis

Two complementary cost-effectiveness analyses will be performed:

i. A within-trial evaluation where cost and health effects of individual participants are limited to the 12-month follow-up period in the trial and
ii. A decision model approach where cost and health effects are modelled to enable the incorporation of longer term benefits and NHS/personal social services (PSS) costs, designed using standard reporting criteria [27].

The estimation of incremental costs and effects, and cost effectiveness ratios will be carried out using the payer’s perspective (NHS England).

Outcomes: Effects will be captured at the individual patient level as part of the multi-centre randomised.

Controlled trial. QALYs will be calculated by attaching available utility weights to the health states generated from the HUI-3 [28] at 12 months post-fitting. Comparisons between the two groups will be corrected for clustering and other baseline characteristics. In an additional investigation, we will investigate the extent to which HHI maps onto HUI-3.

#### Process evaluation: implementation analysis

Semi-structured interviews in FAMOUS will contribute to the process evaluation of the trial and will specifically focus on understanding:

i. Sense making: how the FAMOUS Intervention is understood and compared with existing follow-up and monitoring practices
ii. Implementation: how the FAMOUS Intervention is locally developed and translated into practice
iii. Embedding: the extent to which the FAMOUS Intervention does become incorporated into everyday practices
iv. Integration: the extent to which the FAMOUS Intervention is sustained as part of routine practice

Interviews transcriptions will be thematically analysed using a modified Framework approach [29]. By ‘modified Framework approach’ it is meant that the Framework approach will be initially used to take an inductive approach to theme generation. Subsequent theme refinement will be deductive and guided by the Normalisation Process Theory (NPT). This will produce a matrix of summarised data providing a structure for analysis. This approach will allow us to: (a) answer the specific research questions we have set, whilst (b) allow important insights to be produced inductively. The wider research team, our PPI group and clinical stakeholders will be involved in the analysis process [30].

The implementation analysis (combining interviews and documents) will then:

i. Explore professional perceptions and attitudes towards the FAMOUS Intervention;
ii. Consider initial and enduring challenges to adoption and unintended consequences arising from implementation;
iii. Survey ‘core enabling ingredients’ that must be replicated at other sites, as well as (iv) any capacity for adaptation in context.

A blueprint for implementation for use by those commissioning and delivering services in other settings will then be produced setting out the core barriers and enablers and the costs and/or resources necessary to embed and sustain the intervention in practice.

### Process evaluation: patient experience and acceptability analysis

We will use topic guides informed by the theoretical framework of acceptability [30] to understand participants’ responses to and interactions with their usual care and the intervention to interview participants who fall into a range of daily use/non-use categories (circa 15 per category). We will additionally use the behaviour change wheel [31] to understand the barriers and facilitators to behaviour change not associated with the acceptability of the intervention (i.e. mechanism of impact). Data will be analysed to identify recurrent themes. Inductive thematic analysis will be used followed by deductive thematic analysis using the theoretical framework of acceptability [30] and theoretical domains framework [32]. These analyses will allow us to understand what improvements can be made to the intervention to enhance patient experience and to increase hearing aid use further.

### Methods in analysis to handle protocol non-adherence and any statistical methods to handle missing data {20c}

As described in Section Statistical methods for primary and secondary outcomes {20a}, imputation will be used so that all randomised participants may be included in a sensitivity analysis where appropriate.

### Plans to give access to the full protocol, participant-level data and statistical code {31c}

De-identified participant data will be made available, upon request, in accordance with the NCTU standard operating procedures following the publication of the trial results.

## Oversight and monitoring

### Composition of the coordinating centre and trial steering committee {5d}

The FAMOUS Trial team at the NCTU will have oversight of day-to-day activities of the trial and will be in regular contact with site research teams to check progress and address any queries. The trial team will check incoming routine clinical data for patient enrolment numbers, adherence to the protocol and treatment arm, data consistency and widespread missing data, as per the Monitoring Plan. The trial team, comprising trial management, data management, statistics and programming teams, will meet weekly and on an ad-hoc basis as required.

The TMG consists of the CI, Co-Investigators, Health Economist, PPI representative, Senior Trial Manager, Trial Manager, and Trial Statistician, with other members of the trial team (e.g. Data Manager, IT/Data Coordinator). The TMG will be responsible for the day-to-day management of the trial and will monitor all aspects of the conduct and progress of the trial, ensure that the protocol is adhered to, and take appropriate action to safeguard participants and the quality of data collected in the trial. The TMG will meet monthly and on an ad-hoc basis as required, and will report to the independent TSC.

The TSC will provide overall supervision, monitor progress against targets, and advise the CI and TMG. The TSC consists of independent members, including a clinical Chair, statistician, health economist, behaviour change expert and a PPI member. The CI is also a non-independent member of the TSC. Non-independent observers attend TSC meetings, including a Sponsor representative, the NCTU senior lead, and members of the trial management and statistical team. The TSC will meet initially to review and agree to the protocol, and then approximately every 6 months. Additional meetings may take place if required.

### Composition of the data monitoring committee, its role and reporting structure {21a}

An independent DMC will review unblinded trial data on a minimum annual basis. Additional meetings may be called at the request of the DMC or TSC chair. The DMC consists of independent members, including a clinical Chair and two statisticians, who attend open and closed meetings. Non-independent observers attend DMC open meetings, including the CI, members of the trial management and statistical team. The unblinded trial statistician will attend the closed DMC meetings alongside the independent members. The DMC will operate in accordance with the trial-specific charter, filed in the electronic TMF. Reports will be supplied in confidence to an independent DMC, which will be asked to give advice on whether the accumulated data from the trial, together with the results from other relevant research, justifies the continuing recruitment of further patients using the stop/go criteria. The DMC will report directly to the TSC, who will convey the findings of the DMC to the funder, Sponsor, and regulatory authorities as applicable.

### Adverse event reporting and harms {22}

The occurrence of adverse events as a result of participation within this trial is not expected because the intervention reflects best clinical practice; therefore, no adverse event data will be collected. Inadvertent distress caused by participation in the trial will be reported by the site PI or trial participant to the FAMOUS Trial Management team.

### Frequency and plans for auditing trial conduct {23}

An ongoing review of trial conduct will be undertaken by the trial management team as per the trial monitoring plan. Audits may be carried out by the sponsor or NCTU quality assurance team in accordance with their local auditing plans.

### Plans for communicating important protocol amendments to relevant parties (e.g. trial participants, ethical committees) {25}

All amendments made to the trial protocol will undergo review and approval by the Sponsor, Research Ethics Committee and Health Research Authority prior to implementation. Updated versions of the protocol will be shared with recruiting centres via email and uploaded to the trial website. Any substantial amendments made will also be communicated to the trial registry.

### Dissemination plans {31a}

The dissemination of the FAMOUS trial findings will be via a publication in the NIHR Journals Library, publication in peer-reviewed journals, and presentations at national and international conferences. We will partner with relevant professional bodies to ensure that our findings and recommendations are included in practice guidelines and to identify best practice for implementation and roll out. Recommendations will be shared with NICE based on the trial findings. During the trial, press releases may be issued from NCTU or the Sponsor. Presentations or other material prepared by local investigators to publicise the trial must be reviewed by the CI and NCTU. No party will be entitled to submit any publicity material without prior approval from NCTU. We will create animated videos that will be translated into four key languages, explaining the results in clear terms. These videos will be available on the trial and Biomedical Research Centre (BRC) websites and will be promoted by the RNID (Royal National Institute for Deaf People), our PPI group, and the BRC, to ensure the results are disseminated as broadly as possible.

## Discussion

FAMOUS is, for the first time, creating links between clinical audiology services and research and development in many NHS Trusts. Most healthcare professionals working in NHS audiology services will have little to no research experience, yet their role in embedding research into clinical practice is critical. Therefore, not only will FAMOUS potentially provide evidence to change clinical audiology practice, but it will provide important lessons for future research delivery.

FAMOUS was designed and funded prior to the COVID-19 pandemic. As such, the set up has been delayed until 2022 and recruitment until Spring 2023. We anticipate that some of the UK NHS adult audiology services that expressed an interest in participating in the trial during the grant application phase will likely be unable to take part in the trial. This is due to the COVID-19 pandemic impacting directly on audiology staffing levels and many services removing their provision of follow-up care for new adult hearing aid users. We also anticipate that services will be reduced in the number of new hearing aid fittings they fulfil post-COVID-19, with (i) NHS audiology services having reduced their clinic capacity, and (ii) the Any Qualified Provider Scheme (AQP) directing a proportion of NHS hearing aid fittings out to high street providers. Furthermore, the trial has been designed to enrol NHS services using Auditbase, as this software enables extraction of the routine clinical data for the trial; however, it is known that around 33% of all NHS clinics have not yet migrated to Auditbase, reducing the pool of potential services eligible to participate.

There have been some amendments to the trial design since the initial ethical approval, which includes:

- The introduction of a consent form for participants and significant others, as the CAG recommended this as opposed to the original design to assume implied consent when questionnaire data is returned from participants.
- The option to re-consent a participant if they do not complete their consent form correctly on their first attempt.
- The name of the intervention arm was changed from Enhanced Care to Structured Care which was decided to be more neutral language, removing any implication of bias that the intervention arm is delivering ‘better’ care to patients.

## Trial status

At the time of submission of manuscript revisions (August 2025), 11,844 patients have been included in the trial, with 8,134 eligible to receive their first questionnaire pack at 12 weeks post-hearing aid(s) fitting. 1,711 (21%) participants have consented and provided questionnaire responses at 12 weeks post-fitting. 759 (73%) have provided primary outcome data. Clinic recruitment ended on 7 March 2025, with final primary outcomes to be collected around May 2026.

## Data Availability

Data sharing is not applicable to this article as no datasets were generated or analysed in preparation of this publication. De-identified participant data will be made available, upon request, in accordance with the NCTU standard operating procedures following the publication of the trial results.

## Abbreviations

AQP: Any Qualified Provider Scheme
BRC: Biomedical Research Centre
CAG: Confidentiality Advisory Group
CI: Chief Investigator
COM-B: Capabilities, Opportunities, Motivations and Behaviour
CONSORT: Consolidated Standards of Reporting Trials
COSI: Client Oriented Scale of Improvement
CRCT: Cluster randomised controlled trial
CSRI: Client Service Resource Inventory
DMC: Data Monitoring Committee
DMP: Data Management Plan
FAMOUS: Follow-up and monitoring of new users of NHS hearing aids
HHI: Hearing Handicap Inventory
HUI-3: Health Utilities Index 3
ICC: Intra-class correlation coefficient
ID: Identifier
IOI-HA: International Outcome Inventory for Hearing Aids
ISRCTN: International Standard Randomised Controlled Trial Number
NCTU: Nottingham Clinical Trials Unit
NHS: National Health Service
NICE: The National Institute for Health and Care Excellence
NIHR: National Institute for Health and Care Research
NPT: Normalisation Process Theory
PI: Principal Investigator
PPI: Patient public involvement
PROMs: Patient-reported outcome measures
PSS: Personal social services
QoL: Quality-of-life
QR: Quick-response
REDCap: Research Electronic Data Capture
RCT: Randomised controlled trial
RNID: Royal National Institute for Deaf People
SAP: Statistical Analysis Plan
SIV: Site Initiation Visit
SOS-HEAR: Significant Other Scale for Hearing Disability
SWAT: Study Within A Trial
TMF: Trial Master File
TMG: Trial Management Group
TSC: Trial Steering Committee
UK: United Kingdom
